# Early introduction oral immunotherapy for IgE‐mediated cow's milk allergy: A follow‐up study confirms this approach as safe and appealing to parents

**DOI:** 10.1002/iid3.447

**Published:** 2021-05-18

**Authors:** Laura Badina, Laura Levantino, Valentina Carrato, Giulia Peruch, Fulvio Celsi, Egidio Barbi, Irene Berti, Giorgio Longo

**Affiliations:** ^1^ Department of Pediatrics, Institute for Maternal and Child Health IRCCS “Burlo Garofolo” Trieste Italy; ^2^ Department of Medical, Surgical and Health Sciences University of Trieste Trieste Italy; ^3^ Department of Pediatrics “Santa Maria degli Angeli” Hospital Pordenone Italy

**Keywords:** cow's milk allergy, desensitization, early introduction oral immunotherapy, tolerance

## Abstract

**Introduction:**

Early introduction oral immunotherapy (E‐OIT) in the first year of life can be a safe treatment for infants with cow's milk allergy (CMA). Once the protocol is completed, doubts remain whether children achieve tolerance or remain desensitized. According to current guidelines, this is determined by an avoidance period followed by a re‐exposure to the food allergen during an in‐hospital oral food challenge (OFC). In real life, this approach can be complicated, time‐consuming, and anxiety‐provoking for parents. We assessed the long‐term safety of E‐OIT for CMA in a cohort of children who switched to an unrestricted diet without testing the achievement of tolerance at the end of the OIT protocol.

**Materials and Methods:**

We performed a descriptive analysis of the clinical follow‐up of a cohort of children diagnosed with IgE‐mediated CMA and undergoing E‐OIT protocol in their first year of life. In a previous publication, the same cohort of patients had been studied to assess the feasibility of E‐OIT for CMA. In the present study, we reported the results of a telephone survey, carried out through a questionnaire to their families enquiring about milk consumption and other ongoing atopic conditions of children.

**Results:**

After an average of 4 years from the start of E‐OIT, 62/73 patients (85% of the historical cohort) participated in the survey. Among them, all 56 patients who had previously successfully completed the protocol reported an unrestricted cow's milk intake. Ninety–three percent of these children did not experience any further allergic reactions, while the remaining 7% described only mild and transitory reactions until the 6‐month period after the end of the protocol.

**Conclusions:**

This study confirmed the long‐term safety of E‐OIT for CMA and challenged the paradigm of the need for allergen food withdrawal to discern between desensitization and tolerance. It could be a starting point for planning future trials on this issue.

## 1 INTRODUCTION

Recent studies have described populations of infants diagnosed with IgE‐mediated cow's milk allergy (CMA) undergoing early introduction oral immunotherapy (E‐OIT). The vast majority of these patients successfully introduced cow's milk (CM) along with dairy products in their diet, consuming them regularly over an average time of about 6 months and with no significant adverse effects.^1^, ^2^, ^3^, ^4^

According to current guidelines, the acquisition of tolerance by OIT is defined as the lack of any allergic reaction after a period of avoidance followed by a re‐exposure to the culprit food allergen during an in‐hospital oral food challenge (OFC).^5^ On the contrary, the absence of allergen‐induced reactions during an intake of the food allergen on a regular basis is defined as desensitization.

Therefore, some doubts remain whether children achieve tolerance or remain only desensitized, once the protocol of E‐OIT is completed. Despite differentiating between desensitization and tolerance is considered crucial for identifying who is at risk for further allergic reactions, no studies have so far determined a desirable/required duration of the OIT maintenance phase to develop a state of sustained unresponsiveness which is not reliant on ongoing allergen exposure.^6^

From a pragmatic perspective, we suggest that a change of thinking in this approach could be considered outside of strict research protocols. Remarkably, CM is so widespread in western cuisine that an involuntary full and sustained avoidance is unlikely in real life.

For this reason, in infants undergoing E‐OIT for CMA, we chose not to verify the achievement of tolerance through a period of CM avoidance followed by an OFC at the end of E‐OIT protocol. In this way, we targeted to reduce the risk of bringing patients back to a previous level of allergic reactivity and restarting oral immunotherapy from a milk dose lower than that previously gained.

This study aimed to verify the long‐term safety of E‐OIT in a group of infants who had not been tested for tolerance at the end of the protocol but switched directly to an unrestricted diet.

## 2 MATERIALS AND METHODS

We present a descriptive analysis of the clinical follow‐up of a cohort of infants diagnosed with CMA and undergoing E‐OIT in their first year of life, to assess the incidence of allergic reactions to CM at an average of 4 years from the start of the early introduction OIT protocol.

### 2.1 Background study and cohort history

The same patients' cohort had been previously studied to investigate the feasibility of the E‐OIT protocol for the treatment of IgE‐mediated CMA in an uncontrolled longitudinal study.^3^ The study had involved 73 children under the age of 12 months with IgE‐mediated CMA, admitted to the Allergy and Asthma department of the Institute of Maternal and Child Health, Trieste, Italy, from March 2015 to June 2017. In particular, CMA was diagnosed as IgE‐mediated based on the following criteria, according with DRACMA guidelines^7^:

(i). A history of generalized allergic reactions in one or more organs (such as urticaria/angioedema, vomiting, or respiratory symptoms) occurred within 2 h after CM ingestion.
(ii). A skin prick test positive for fresh CM (wheal size larger than or equal to 5 mm) and at least one of the main CM proteins between casein, beta‐lactoglobulin, and alfa‐lactalbumin (wheal size larger than or equal to 3 mm).
(iii). Specific IgE levels (ImmunoCAP® Phadia AB) for CM and at least one main CM protein higher than 0.35 kU/l.

Were excluded from the study: children under three months of age, children with non‐IgE‐mediated clinical manifestations, and children with a known immunodeficiency.

To test eligibility for OIT, infants were subjected to a low‐dose oral food challenge (OFC), with the administration of three increasing doses (1, 5, and 10 ml) of CM every 30 min. During the OFC, IgE‐mediated clinical manifestations were recorded according to Clark's classification.^8^ The low‐dose OFC was performed to find out a safe dose of CM with which to start OIT. Therefore, were excluded patients experiencing severe anaphylaxis (a Class 5 reaction) at any CM dose and, for the difficulty to identify a safe dose to be continued at home, also patients reporting generalized allergic manifestations (Class ≥ 2 reactions) at the first dose of 1 ml of CM.

Every eligible infant followed a home oral immunotherapy protocol, with periodic increases of the milk dose, initially tested in hospital. Once the target dose of 150 ml of CM in the same meal was reached during the OIT, we taught parents to offer their children milk in quantities and varieties they liked, so that gradually reduced the need to offer milk regularly in pre‐established times and doses.

Of the 73 children enrolled in the baseline study, 68 (93%) started the E‐OIT protocol: among them, 66 (97%) reached the CM target dose, that was a daily intake of 150 ml of milk or an equal dose of dairy, in a mean time of 6 months. These 66 patients, during the last clinical visit at the end of the protocol, were prescribed an unrestricted daily intake of CM or dairy products (given ad libitum). The remaining seven patients (10%) were not considered suitable for OIT or had to discontinue OIT due to persistent allergic reactions (mainly vomiting).

The only atopic comorbidity of children screened in the baseline study was atopic dermatitis, which affected 58% of them.

### 2.2 Current study

In this long‐term follow‐up study, the whole cohort of patients was involved in a survey, in which outcome data were collected up to December 2019 through a telephone interview with their families.

A follow‐up questionnaire, designed by researchers of University of Trieste, was administered to determine the current type of diet of children (CM‐free vs. unrestricted diet and, in this case, frequency and quantity of CM and/or dairy products intake) and to investigate CMA recurrence after the E‐OIT protocol or CMA persistence without an E‐OIT approach.

Information was also obtained about the evidence of other food allergies (still present or outgrown) and the presence of other atopic diseases.

## 3 RESULTS

A total of 62 out of the 73 patients of the historical cohort (85%) responded to the telephone survey, participating in the follow‐up study: children's median age was 4 years old and 68% were males. Among these 62 patients: five had not been passed through an E‐OIT attempt, due to generalized allergic reactions triggered by 1 ml of CM at the low‐dose OFC, while 57 had started E‐OIT. Of them, 56 had successfully completed the protocol, and one had dropped out for persistent reactions during the protocol.

**Figure 1:**
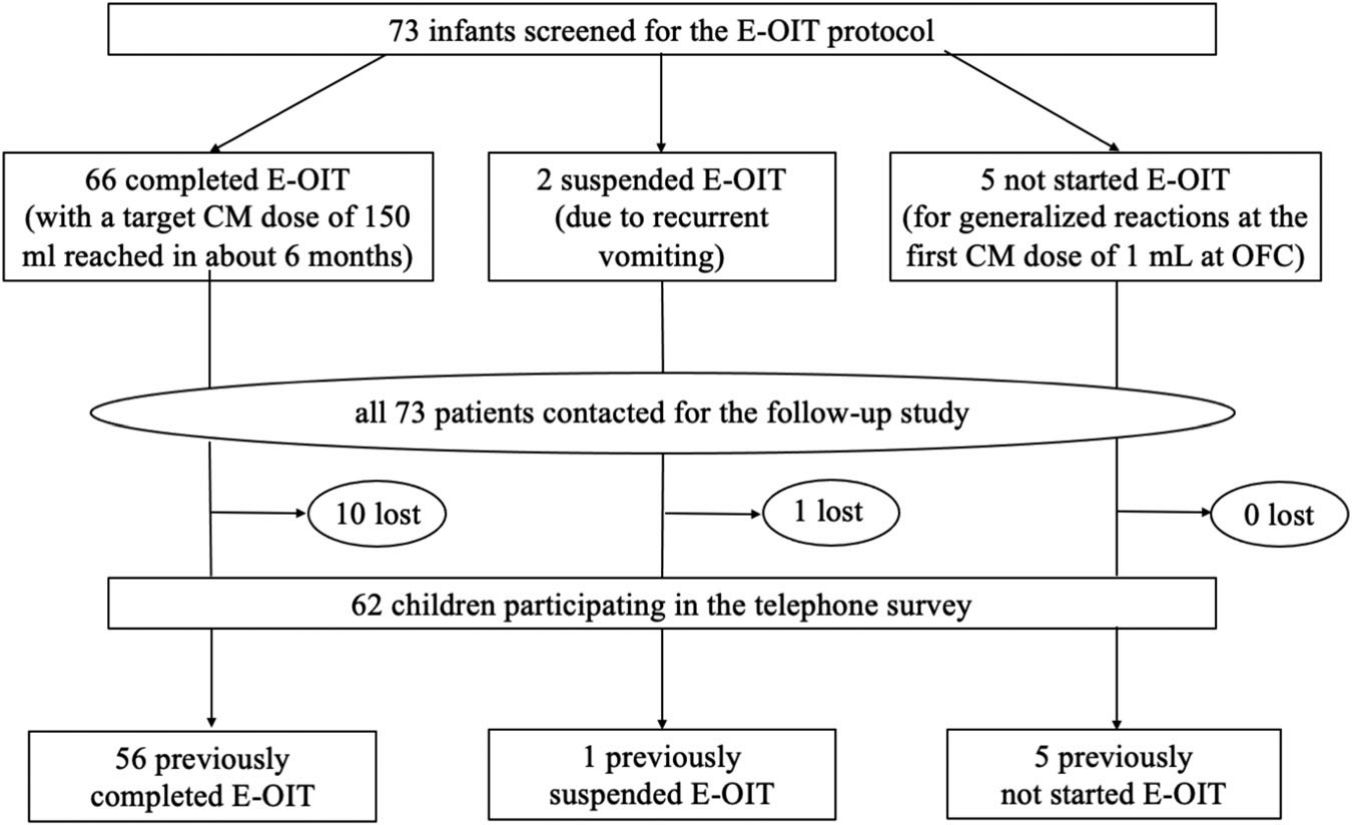
In a previous uncontrolled longitudinal study, 73 infants (under 12 months of age) were screened to test the feasibility of the E‐OIT protocol for the treatment of lgE‐medicated CMA. Among them: 66 completed E‐OIT; 2 suspended E‐OIT; and 5 did not start E‐OIT. Four years after the start of the E‐OIT protocol, the same cohort of patients was contacted for the present follow‐up study. A total of 62 children of the historical cohort participated in the telephone survey. CMA, cow's milk allergy; E‐OIT, early introduction oral immunotherapy

Figure 1 shows the groups of patients participating in the follow‐up survey with reference to the historical study.

All of the 56 cases (100%) who had completed E‐OIT reported an unrestricted CM intake. Based on the last 2‐week food diaries, these children consumed an average daily CM dose of 250 ml, equal to a daily intake of 8.3 g of milk proteins (~0.5 g/kg/die). Fifty‐two of them (93%) did not experience any further allergic reactions; the remaining four patients (7%) described CM‐related reactions until the 6‐month period after the end of the protocol. All these reactions were listed as mild and transitory, without need for medical intervention.

The only child who had at the time dropped out of the E‐OIT protocol gradually reintroduced CM at home with no problems.

Among the five children initially ineligible for OIT, three were still on an exclusion diet, while two reported an unrestricted intake of CM at the follow‐up: one of them after a second successful OIT, and the other one after a gradual CM introduction attempted at home by parents on their own initiative.

Regarding the evolution of atopic march: atopic dermatitis persisted in 37% of patients (against 58% at the baseline), while other allergies (absent at baseline) developed in 16% of children. They were both food allergies (eggs, wheat, and nuts) and inhalant allergies (mite and pollens).

Table 1 shows the details about the baseline study's and the follow‐up study's patients and outcomes.

**Table 1:** Details of patients and outcomes of the baseline study and the follow‐up study

	Baseline study cohort^3^	Follow‐up study cohort (Median duration, months: 39, IQR 32–47)
Number of patients	73	62
Male patients, number (%)	50 (68)	42 (68)
Median age, years (IQR)	0.6 (0.2–0.9)	4 (2.8–4.7)
E‐OIT, number (%)		
Successfully completed	66 (90)	56 (90)
Suspended for persistent reactions	2 (3)	1 (2)
Not started for ineligibility	5 (7)	5 (8)
Type of diet, number (%)		
Unrestricted	66 (90)	59 (95)
of which completed E‐OIT	59	56
suspended E‐OIT	2	1
not started E‐OIT	5	2
CM‐free diet, number (%)	7 (10)	3 (5)
of which completed E‐OIT	0	0
suspended E‐OIT	2	0
not started E‐OIT	5	3
Presence of atopic dermatitis, number (%)	42 (58)	23 (37)
History of other food allergies, number (%)	0 (0)	10 (16)
Manifestation of inhalant allergies, number (%)	0 (0)	6 (10)

Abbreviations: E‐OIT, early introduction oral immunotherapy; IQR, interquartile range.

## 4 CONCLUSIONS

This study confirmed the long‐term safety of E‐OIT for CMA of other reports.^1^, ^2^, ^4^ Besides, it challenged the paradigm of the need for CM withdrawal to discern between desensitization and tolerance. In real life, this approach can be complicated, time‐consuming, and anxiety‐provoking for parents. Moreover, as far as cow's milk proteins are concerned, the possibility that desensitized patients accidentally suspend a protracted unrestrictive diet is so limited that it appears to make limited sense.

Remarkably, by excluding from OIT children with generalized reactions triggered by 1 mL of CM, as well as possibly children with severe anaphylaxis at any dose, during the low‐dose OFC, we had selected a population of infants with CMA at low to moderate risk. In this perspective, E‐OIT, without a period of allergen absence, might mimic, in a sort of secondary prevention, what had happened in the cohorts of the studies that assessed the efficacy of early introduction of allergenic food in reducing the incidence of food allergy.^9^, ^10^, ^11^

This article limits were the lack of a randomized control group, a complete OFC at entry, a final dosage of cow's milk specific IgE and IgG4 levels.

In any case, we aimed to report a pragmatic experience on a still little explored issue, concerning a significant number of patients and an extended period of follow‐up.

Since we had excluded infants reactive to a very small amount of CM (1 ml), we could not extend our results to this population, theoretically at higher risk of severe anaphylaxis during OIT. Nevertheless, in Bonè Calvo et al.^4^'s study on a larger population of infants with CMA, in which no one had been in advance excluded from OIT, no significant adverse reactions occurred, and the protocol was successfully concluded by 98% of participants.^4^

Further long‐term data are needed to confirm that tolerance achieved by early desensitized infants is as reliable as that achieved by children who spontaneously resolve their food allergy, and to define the adequate duration of OIT to move them safely towards an unrestricted diet. This report could represent a starting point for planning future trials on this issue.

## CONFLICT OF INTERESTS

The authors declare that there are no conflict of interests.

## ETHICS STATEMENT

The Independent Bioethics Committee of the Institute for Maternal and Child Health‐ IRCCS Burlo Garofolo of Trieste (Italy) approved the protocol. All parents or legal guardians signed written informed consent for minors' participation.

## AUTHOR CONTRIBUTIONS

Laura Badina, Giorgio Longo, Irene Berti, and Egidio Barbi contributed to the article's conception. Giulia Peruch and Celsi Fulvio provided the data. Laura Badina, Laura Levantino, and Valentina Carrato wrote the draft and worked on the critical revision. All the authors read and approved this final version.

## Data Availability

The data that support the findings of this study are openly available in PubMed Central (PMC) at: https://doi.org/10.1016/j.jaci.2010.12.108, reference number [1]. https://doi.org/10.1016/j.anai.2013.09.001, reference number [2]. https://doi.org/10.1111/pai.13057, reference number [3]. https://doi.org/10.1007/s00431-020-03731-3, reference number [4]. https://doi.org/10.1111/all.13319, reference number [5]. https://doi.org/10.1111/j.1398-9995.2005.00882.x, reference number [6]. https://doi.org/10.1097/WOX.0b013e3181defeb9, reference number [7]. https://doi.org/10.1136/adc.88.1.79, reference number [8]. https://doi.org/10.1016/j.jaci.2020.08.021, reference number [9]. https://doi.org/10.1056/NEJMoa1414850, reference number [10]. https://doi.org/10.1016/j.jaci.2015.12.1322, reference number [11].
